# Medical Progress

**Published:** 1856-04

**Authors:** 


### EDITORIAL DEPARTMENT.

### MEDICAL PROGRESS---NEW ERA.

Long since the advent of the writer of this article, who is by no means an old man, a medical school was one of those institutions of which we occasionally read, and perchance, now and then a doctor, who had trod the "halls of Science," was seen and gazed at by the people with as much wonder as Dr. Kane would be now-a-days, by a clod hopper, whose utmost knowledge of navigation was the mode of guiding a Buckeye canoe across the current of a swollen creek. A man who could cut a leg or an arm off and "heal it up," was regarded as scarcely less than a miracle man, and one who could explore with the knife, and extract foreign bodies from hidden organs successfully, almost a demigod. The names of Dudley and Drake were justly revered as the embodiment of medical science in the West; orbs from whom the healing rays radiated through the wilderness around them.

Heroes indeed, were the students who undertook and completed the pilgrimage to those Æsculapian temples. The difficulties opposing the attainment of medical information, but added dignity to its votaries. Meagre were the appliances for teaching in the best of our medical schools, poor indeed those of our western institutions, which numbered but one or two at the time alluded to. Now and then a professor crossed the broad waters and gathered what could be found in the schools and hospitals of another continent. From them, students, by a long journey through the wilderness, heard the results of their observation and experience.

These venerable pioneers did not think it essential that they should claim the facilities for teaching, which older and richer institutions had, but modestly imparted all that the circumstances enabled them to do. They taught, and taught satisfactorily, the plain principles of medicine, though they may have been deficient in the means of illustration.

But things have most marvelously changed since then. Our territory has spread from ocean to ocean. The wild wilderness falls before the rolling tide of emigration, and blooms and blossoms as the rose, in its wide wake. Towns and cities spring, with almost magic quickness, from the ground; the mountains are leveled or bored by the sturdy hand of enterprise. The iron horse runs with resistless speed his course, wherever he listeth. All-powerful steam, with its iron arm, the mighty servant of commerce, reciprocally interchanges the products of the most widely separated regions; the lightning carries our messages, and we whisper in the ear of our friend at a thousand miles distance as quickly as to the one on our bosom; the press, the eyes and ears of the nation, talks at every fireside; books, professional and miscellaneous, speak to us of science and literature; magazines and pamphlets, blue, yellow, red, and white, furnish small enjoyment for the light and superficial. Infinite indeed, are the masses of advancement.

It were strange if medical schools should not make some progress in the midst of such onward activity. Nothing can now stand still—all must move or be run over. These schools have multiplied and progressed generally, with the things around them. Almost every State, old and new, has now its school or schools of medicine, and every town, village, and neighborhood, its doctors with their "sheep skin cataplasms."

The tyro cuts away fearlessly and often successfully. But today we read of "surgery in Illinois," where a crushed leg was amputated with a butcher's knife whetted on a brick-bat, the arteries taken up with a crooked fork, the bone divided with a handsaw, the wound dressed with a cotton rag, stuck on with shoemaker's wax, and eight inches of whiskey as an anesthetic. Such are the triumphs of progressive surgery; and practical medicine might urge a thousand illustrations of her wonderful resources.

But notwithstanding such results from the light before us, still more astounding achievements are yet to come. Indeed these astonishing results of the medical teachings of these latter times, are but the dim corruscations of a medical constellation, whose region in the firmament of science has been so far above all which have yet gleamed upon us, that their lengthened rays are but just now guilding the eastern horizon of the world of science. True, they have shone in their own sphere, illuminating the space which time has been given for their light to reach, but like those fixed stars, those worlds of light, whose distance from us is so immense that this dull brown earth is but just now receiving and dimly reflecting their rays, ~~which~~ promise soon to make us shine again with their heavenly lustre. Genius reaching beyond its age in its far-seeing sagacity.

Hear the announcement of this light-giving constellation:

"The Board of Trustees and Faculty of the Iowa Medical College, take pleasure in announcing to the medical profession and the public, that the regular session of this Institution will open in this city, on the first Monday in November next. The Trustees, clothed with the most ample legal powers, have completed the organization of the Medical Faculty, in a manner which cannot fail to satisfy the true friends of Medical Education throughout the country, and the plan of instruction is, for the first time in this State, arrayed in such a manner as to fully realize the ideas and recommendations of the American Medical Association. It is well known, that this body, composed of medical men, from every portion of our widely extended country, has been for several years, laboring to elevate and improve the Medical Literature of the United States. In some respects, their labors have accomplished the desired object; but in the most important particular—a reform in medical teaching—much yet remains to be done.

"It is designed to make the Iowa Medical College an instrumentality, in improving the state of Medical Education in the West, and to place it upon such a footing, as to command the respect of the profession at home, and make it a creditable representative of our Medical Literature abroad.

"No one doubts the need of such an Institution in Iowa, and the extended—we may say, almost universal—sanction of the profession in this portion of the West affords a sure foundation for a brilliant future and inevitable success. In view of this certain prospect, every facility has been accumulated. It is confidently asserted, that the most extensive and perfect means of illustrating the several courses of instruction are provided, and every essential opportunity afforded for a thorough prosecution of medical studies. The finest and most commodious lecture rooms in the State, are being fitted up and the arrangements of the College building, with reference to anatomical pursuits, is unsurpassed."

Such is the pronunciamento of this organization, which is to reform and perfect the medical teaching of the West. Now to lay aside all rhetoric and look to the merits of this enterprise. Certainly the thing is *modest.* We have first and foremost in the Faculty of ten, here announced, at least *seven* who probably, in the capacity of teacher, *never delivered a medical lecture* in their lives. They were men "from every portion of the country, who had been laboring for years to elevate and improve the medical literature of the United States." This may be true, but certainly they have never been very prominently before the medical public, and the evidences of their efforts in the way of reform, prove nothing more than that their merit consisted *in good intentions and obscure worth*, rather than the impression made upon the progress of science. Granting them, however, all the ability claimed, unfortunately, "the plan of instruction for the first time in this State, arranged in such a manner as to fully realize the ideas and recommendations of the American Medical Association," was frustrated, as only *four* of these self-constituted medical decemvirs attempted to enlighten the medical world, during the past winter, of realization of the ideal of the Iowa Medical College. Indeed, the facility with which the Faculty, from time to time, in the space ot a few months, was remodeled by new appointments, to fill vacancies occurring by resignations, &c., shows the number of these qualified medical reformers to be surprisingly large in this region, although we had been led at first to think the *original ten* were all of the "same sort left." Now what are the facts in regard to this institution, which is so much vaunted as the only one in which an education, up with the requirements of the age, can be obtained.

The course was opened by the founder with an introductory, attempting to show the necessity for a new school, the superior advantages of the one he then represented, the old fogy character of the University—and in general, the medical passibilities of the West. The number of students in attendance five or six, at most, which never increased, though "*only nineteen*" is claimed by the valedictorian. Four were graduated—two of whom never attended a course in the institution. The superb halls are situated in the third story of a business house—the Museum not Hunterian, to say the most of it—the chemical apparatus insufficient to illustrate many of the simplest processes, while the hospital departments consisted of half a dozen beds, and notwithstanding "the special attention of a clinical teacher," we fear *few* students left, feeling themselves qualified for the "important responsibilities of the sick room," unless the Faculty has the art of imparting that miraculous power to practice which they claim to possess to teach.

But to pursue the history of this Institution. It has, according to the valedictorian of the Faculty, established a name, (!) notwithstanding "an opposition—a vindictive and fiendish opposition, which has no parallel in the annals of scientific strife." "Treachery, falsehood, and detraction," "base slander," "malicious intrigue," "irony and low wit," "have exerted their force to heap obloquy upon the few who have prosecuted the noblest of sciences within these walls." It is not stated *who* has so malignantly assailed this infant giant, that, like Hercules, has so valorously resisted its poisonous enemy. Very forbearingly, however, these futile attempts at destroying this Appollonian temple; and "all this exhibition of evil passion is left to its own natural operations of decay." They are consoled by the reflection that "the world, the flesh, and the *devil cheer us on.*" "The rest of mankind," then, are their impotent traducers. "Peace and science truly." Under these dreadful difficulties, however, we are still informed that no institution in the country affords more ample means of practical demonstration, in anatomy, clinical practice, surgery, &c. This, it is to be hoped, is, comparatively speaking. A single subject might be ample for a dissecting class of five or six, but not for eighty or a hundred students. A half dozen beds will give us some variety in the way of disease, a dozen operations might afford us an opportunity of seeing something more than an amputation or breaking down a cataract. But are these to be paraded before the public as means equal in extent or variety to "any school in the country?" We might suppose, therefore, that the intention was to say that the wants of a small, very small class were, to some extent, met. But we are driven from this supposition, when the orator turns from the means of teaching, towards the men who have taught. Of their capacity and standing he us gives information—tells us how they have overcome all the obstacles to successsful teaching, and in conclusion, pits the Faculty and College against any other in the western country—and this challenge follows a reference to his own performance as to which he feels "a sum of satisfaction." He glories in the storm which has swept over but did not scathe his College; the ship is tried; the timbers are flexible but strong; the nature of the opposition is "contemptible," but cost several pages of scorn; warns older schools that their doom is fixed; that perpetuity is only the reward of talent, zeal and devotion; based upon these the Iowa Medical College must stand erect amidst the downfall and disasters of already well established schools! We must be charitable and suppose the speaker to look through microscopic glasses of considerable power—or to have inhaled exhilerating gas to inspiration, as did the man, who, under similar influence, exclaimed:

"I'm larger grown from head to tail
Than mammoth elephant or whale;
I feel a tangible expansion,
A semi-infinite dimension.
Earth quake, quiver, quail!
For lo! I stride a comet's tail,
If my deserts you fail to acknowledge,
I drive plump against your college."

But to conclude, we will give an epitome of what have been the actual results of this vaunted school of medicine.

We have alluded to their general arrangements. The course was opened by an introductory attempting to show the necessity of a second school in this city; that they would open with a respectable class, and would ultimately succeed—about one half the Faculty in attendance—and *five students*, three of whom were the private students of the professors. The influence then reached to two or three not immediately, more or less influenced by personal considerations towards the Faculty. True, "nineteen" is claimed, but we challenge the proof of the regular attendance of over six during the course. Four were graduated, I believe, but two of whom, if we are rightly informed, were in regular attendance during the winter. That every feature in the picture, as portrayed by the flourish of the announcement, or painted in the dark shades and bright colors of the valedictory artist, are amazingly exaggerated is palpable.

The truth is, the scheme is one of private spite on the part of its originator; the object to gratify an ambition thwarted in former designs. That there is a necessity for a second school in Iowa cannot be defended by a plausible argument. We desire no monopoly of science, but any one can see, who will take the trouble to trace the origin of this scheme, that it is the child of malign ambition, not the legitimate offspring of public requirement.

It has been sustained by misrepresentations as to its necessity and prospects, and it is now too late to enter the special and peculiar plea of the designing, *persecution.* The thing is too bald, and public sympathy will not be sufficiently melted and warmed by the cry of oppression to nurture, develope and mature the offspring of such parentage. Its parent would willingly see it abort, could he by the failure, destroy the flattering progress of the University, from whose Faculty he was virtually expelled.

For the majority of those whom he has associated with him, from time to time, we have naught but the kindest feelings. They have been unfortunate in tacitly endorsing the charlatanry, pomp, and self laudation which has characterized the circular and the valedictory. We hope never to be found opposing any measure which honestly endeavors to advance the science of our adoption, but always both schemes and men, who for personal gratification, or worse, for malicious purposes towards others, attempt to sanctify, by specious appeals to false calculations and individual feelings, projects which they know and feel are unworthy the true friends of professional dignity and advancement.

This is but a revival of an abortive effort of the same kind a year or two since. It is very well known in our State, that the medical school established here as a department of the University, has met with every reasonable encouragement. This has stung the envious and malicous founder of this great *reforming*

*institution*, and has stirred up the bitter elements of his poisoned blood. He has ever labored to advance his own reputation rather than science. Where these two interests clash, it is no sacrifice with him to trample upon every principle which dignifies the profession, and to endeavor to build a pedestal that might lift his pigmy statue on a level with the elevated worthies who now overshadow him.

We feel that some apology is due many of our readers for obtruding this matter upon them. We generally endeavor to make our Journal of practical use to subscribers, who are practical men in the main. We do not think it very unbecoming, however, to expose charlatanry, though it may originate with Faculties, "clothed with the most ample legal powers." We thought it due, especially to our brethren engaged in teaching medicine, to warn them of the advance of this medical phenomenon, that they might *voluntarily close doors*, and save themselves the obloquy of having to lecture hereafter to medical "halls deserted." Next winter we perhaps ought not to be astonished to see the "upper story" crowded with medical savans, "wise men from the east," to watch the culmination of this fast rising medical luminary; view its vast arrangements, examine its facilities for teaching, and "by authority" go home and "array themselves for the first time in such a manner as to fully realize the ideas of the American Medical Association," by copying the "ways and means" of the Iowa Medical College, at Keokuk, Iowa. A.

